# A rare case of chronic myeloid leukemia with secondary chromosomal changes including partial trisomy 17q21 to 17qter and partial monosomy of 16p13.3

**DOI:** 10.1186/1755-8166-3-6

**Published:** 2010-03-16

**Authors:** Walid Al Achkar, Abdulsamad Wafa, Hasmik Mkrtchyan, Faten Moassass, Thomas Liehr

**Affiliations:** 1 Molecular Biology and Biotechnology Department, Human Genetics Division, Atomic Energy Commission, Damascus, Syria; 2 Jena University Hospital, Institute of Human Genetics and Anthropology, Jena, Germany; 3 Department of Genetic and Laboratory of Cytogenetics, State University, Yerevan, Armenia

## Abstract

**Background:**

The so-called Philadelphia (Ph) chromosome is present in almost all cases with chronic myeloid leukemia (CML). Around 5-10% of these patients show complex translocations involving other chromosomes in addition to and/or besides chromosomes 9 and 22. As nowadays most CML cases are treated with Imatinib, variant rearrangements have in general no specific prognostic significance, though events of therapy resistance remain to be studied.

**Results:**

Here we report a Ph chromosome positive patient with hematological typical chronic phase CML. Untypically, an unbalanced complex rearrangement involving chromosomes 16 and 17 leading to a deletion of 16pter and partial trisomy of 17q21 to 17qter, was identified besides a trisomy 8 and an additional Ph chromosome in a part of malignant cells.

**Conclusion:**

Here a novel and cytogenetically unique case of a Ph chromosome positive CML clinically in chronic phase is reported, having complex secondary chromosomal aberrations. Thus, CML patients with complex chromosomal changes are nonetheless treatable by Imatinib.

## Background

Chronic myeloid leukemia (CML) is a clonal malignant disorder of a pluripotent hematopoetic stem cell characterized by the presence of the Philadelphia (Ph) chromosome in more than 90% of patients. The Ph chromosome is a product of the reciprocal translocation t(9;22)(q34;q11), which transposes the 3' portion of the ABL oncogene from 9q34 to the 5' portion of the BCR gene on 22q11.2. The crucial pathogenetic consequence of this translocation is the creation of a chimeric BCR/ABL gene on the derivative chromosome 22 [1]. The expression of the BCR/ABL chimeric protein with an increased tyrosine kinase activity plays an essential role in the pathogenesis of CML [2].

The progression of CML from chronic phase (CP) to blast crisis (BC) is frequently associated with nonrandom secondary chromosomal aberrations such as +8, i(17q), +19 and an extra Ph chromosome [3]. Imatinib mesylate (Glivec, formerly STI571) was designed specifically to inhibit the tyrosine kinase activity of the BCR/ABL protein and other tyrosine kinases such as cABL, c-KIT and PDGF (platelet-derived growth factor receptor). By binding to an active site of the tyrosine kinase, Glivec switches off downstream signaling, cells stop proliferating and apoptosis ensues [4]. Many studies have shown a high efficiency of Imatinib therapy to achieve a complete or major cytogenetic response, i.e. a reduction to 0-34% Ph-positive cells. This positive effect may be attained in cases with a simple t(9;22), in such with complex translocations resulting in a BCR/ABL fusion gene, as well as in cases with cytogenetic clonal evolution [5,6].

Herein we report a rare case of a Ph chromosome positive CML with a derivative chromosome 16 leading to a partial trisomy 17q21 to 17qter and partial deletion of 16pter, which was nonetheless successfully treatable by Imatinib.

## Case report

A 30-year old male was diagnosed to suffer from CML in CP after a blood cell count was initiated in June 2009 due to splenomegaly and severe loss of weight. The patient was treated with Imatinib mesylate at a 400 mg/day for overall 12 months and previous relevant symptoms disappeared. Hematologic parameters were as follows: hemoglobin 129 g/l; platelets count was 103 × 10^9^/l; and white blood cells (WBC), 5 × 10^9^/l with 57.2% neutrophils, 36.3% lymphocytes, 4.8% monocytes, 1.3% eosinophiles and 0.4% basophiles.

**Figure 1:**
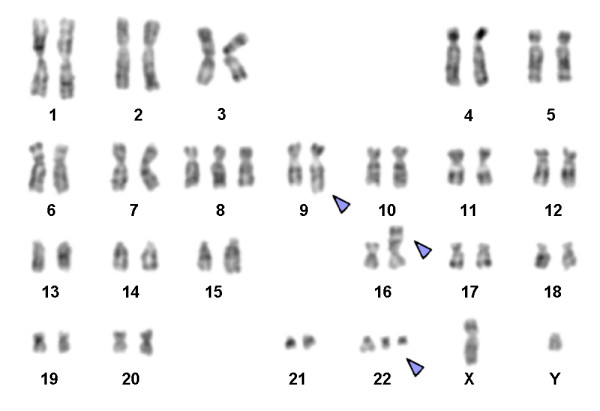
**GTG-banding revealed a complex karyotype involving two further chromosomes besides chromosomes 9 and 22**. All derivative chromosomes are marked by arrowheads. Karyotype: 48,XY,+8,t(9;22)(q34;q11),der(16),t(16;17),+der(22).

**Figure 2:**
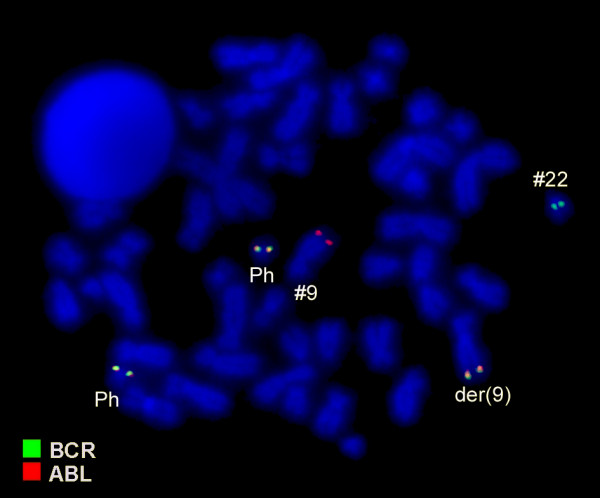
**Fluorescence in situ hybridization (FISH) using probes for BCR (green) and ABL (red) showed the presence of two Ph-chromosomes**. Abbreviations: # = chromosome; der = derivative chromosome; Ph = Philadelphia-chromosome.

**Figure 3:**
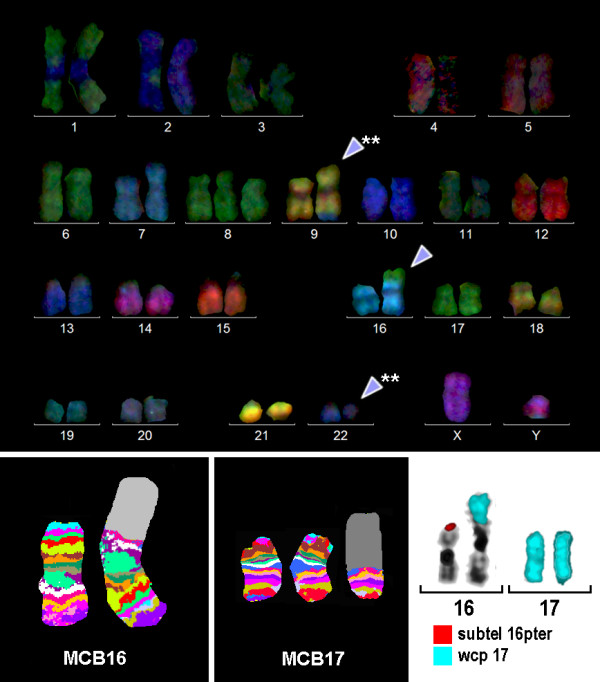
**Karyotype and chromosomal aberrations were confirmed using molecular cytogenetic approaches**. (A) M-FISH confirmed the complexity of the karyotype: 47,XY,t(9;22),der(16)t(16;17),+8. (B) and (C) The application of MCB 16 and 17 revealed the chromosomal breakpoints on the corresponding derivative chromosomes as 16p13.3 and 16 q22 and 17q21. (D) The deletion of the subtelomeric region on the der(16) and the translocation of chromosome 17 material was also demonstrated by the application of a subtelomeric probe for 16p (Abbott Molecular/Vysis, USA) and a whole chromosome painting (wcp) probe for chromosome 17.

Karyotyping was done after initiation of chemotherapy treatment, showing the following karyotypic changes. A complex karyotype 48,XY,+8,t(9;22)(q34;q11),der(16),t(16;17),+der(22)/47,XY,+8,t(9;22)(q34;q11),der(16),t(16;17)/46,XY,t(9;22)(q34;q11) was determined in GTG-banding (Fig. 1) and further specified by molecular cytogenetic studies (Fig. 2, 3). Dual-color-FISH using a probe specific for BCR and ABL revealed a typical Philadelphia-chromosome with BCR/ABL fusion gene was present and the second derivative chromosome 22 was a second Ph chromosome (Fig. 2). M-FISH, was applied to exclude further cryptic rearrangements (Fig. 3A). As no other additional changes were found by that approach, array-proven high-resolution multicolor banding (aMCB) (10) probe sets for chromosomes 16 and 17 were applied. Thus, an intrachromosomal rearrangement on the derivative chromosome 16 was detected additionally to the translocation of chromosome 17 material to the derivative chromosome 16 (Figs. 3B and 3C). Finally, a subtelomeric probe for 16pter revealed a partial deletion on the derivative chromosome 16 (Fig. 3D). Thus, the following final karyotype was obtained: 48,XY,+8,t(9;22)(q34;q11),der(16)t(16;17)(16qter→16q22::16p13.3→16q22::17q21→qter),+der(22)t(9;22)(q34;q11)[8]/47,XY,+8,t(9;22)(q34;q11),der(16)t(16;17)(16qter→16q22::16p13.3→16q22::17q21→qter)[8]/46,XY,t(9;22)(q34;q11)[4].

## Discussion

In a case of a CP Ph chromosome positive CML additional chromosomal changes were detected. Apart from a trisomy 8 and an additional derivative chromosome 22 two additional chromosomal alterations were present: an intrachromosomal rearrangement involving 16q22, 16p11.3 and an interchromosomal one with breakpoints in 16q22 and 17q21. To our knowledge, this translocation has been never observed in CML before [7].

In 5-10% Ph chromosome CML cases have complex translocations in addition to those and/or besides chromosomes 9 and 22 [1]. At present it appears that in such rearrangements any other chromosome may be involved. However, it has been suggested that distribution of chromosomes and breakpoints is non-random with the chromosomal bands most susceptible to breakage being 1p36, 3p21, 5q31, 6p21, 9q22, 10q22, 11q13, 12p13, 17p13, 17q21, 17q25, 19q13, 21q22, 22q12 and 22q13 [8], showing one match with the present case, i.e. 17q21.

Additionally, the two breakpoints in chromosome 16 have previously been reported to be associated with acute nonlymphocytic leukemia (ANLL) type M4 [9]. 16q22 is frequently found in the ANLL typical inversion (16)(p13q22) and the related interchromosomal translocation (16;16)(p13;q22). These chromosome 16 rearrangements resulted in the disruption of the myosin heavy chain (MYH11) gene mapping to chromosome 16p13.13 to 16p13.12 [10]. MYH11 encodes the smooth-muscle myosin heavy chain and belongs to the family of conventional myosins. The well-characterized biological function of myosins is their ability to use the energy of ATP hydrolysis to move actin filaments and produce muscle force. Recently, myosins have been implicated in a variety of other intra-cellular functions, including cell migration, adhesion, control of cell shape, and membrane traffic but are implicated also in a variety of other cellular functions, many of which are relevant for cancer formation [11]. Unfortunately, in the present case no material was available to test if MYH11 was involved in the rearrangement.

During CML progression trisomy of chromosomes 8 and a second Ph chromosome are frequent anomalies in CML [3]. Also, another leukemogenic effect of the der(16) may be due to increase copy number of the 17q21 to 17qter region. Morerio et al. [12] have reported that trisomies for this region represent a negative prognostic indicator in myeloid neoplasia. Also an isochromosome 17q is a well-known progression step in CML.

In conclusion, here we reported a novel and cytogenetically unique case of a Ph chromosome positive CML in CP. Especially the latter fact being still a CML in CP and not in BC when having such complex secondary chromosomal aberrations is intriguing. We suggest that the reported patient had nonetheless such a good response to Imatinib as irrespective of complex chromosomal changes he was treated 'just in time' before further mutations appeared leading to BC.

## Materials and methods

### Chromosome analysis

Chromosome analysis using GTG-banding was done according to standard procedures [13]. 20 metaphases analyzed from unstimulated bone marrow culture were analyzed. Karyotypes were described according to the International System for Human Cytogenetic Nomenclature [14].

### Molecular cytogenetics

Fluorescence in situ hybridization (FISH) using LSI BCR/ABL dual color dual fusion translocation probe (Abbott molecular/Vysis, USA) and subtelomeric probe for 16pter (Abbott molecular/Vysis, USA) were applied according to manufacturer's instructions together with a whole chromosome painting (WCP) probe for chromosome 17 [15]. Array-proven multicolor banding probe (aMCB) sets based on microdissection derived region-specific libraries for chromosome 16 and 17 were applied as described [16] 20 metaphase spreads were analyzed, each using a fluorescence microscope (AxioImager.Z1 mot, Zeiss) equipped with appropriate filter sets to discriminate between a maximum of five fluorochromes and the counterstain DAPI (Diaminophenylindol). Image capturing and processing were carried out using an ISIS imaging system (MetaSystems, Altlussheim, Germany) for the evaluation aMCB.

## Competing interests

The authors declare that they have no competing interests.

## Consent

Written informed consent was obtained from the patient for publication of this case report and accompanying images. A copy of the written consent is available for review by the Editor-in-Chief of this journal.

## Authors' contributions

AW and FM performed the cytogenetic studies in the present case and collected the data relative to this case report. WA supervised the cytogenetic analysis as Director of the HGD. HM, AW, FM TL did the molecular cytogenetic analysis and interpretation. TL drafted the paper and all authors contributed to the finalizing of the manuscript and read and approved it also in its final version.
